# The complete mitochondrial genome of *Chibiraga houshuaii* (Lepidoptera, Limacodidae) and its phylogenetic implications

**DOI:** 10.1038/s41598-024-57709-4

**Published:** 2024-03-25

**Authors:** Yanpeng Cai, Aihui Yin

**Affiliations:** Molecular Diagnostic Research Center, Guizhou University of Traditional Chinese Medicine, Guiyang, 550025 China

**Keywords:** Evolutionary biology, Entomology, Phylogenetics, Mitochondrial genome

## Abstract

*Chibiraga* is a mall East Asian genus in the family Limacodidae (slug-moths). The latter includes many agricultural pests. Mitochondrial genome analysis is an important tool for studying insect molecular identification and phylogenetics. However, there are very few mitogenome sequences available for Limacodidae species, and none for the genus *Chibiraga* at all. To explore the mitogenome features of *Chibiraga* and verify its phylogenetic position, the complete mitogenome of *Chibiraga houshuaii* was sequenced and annotated. The complete 15,487 bp genome encoded 37 mitochondrial genes, including 13 protein-coding genes (PCGs), 22 transfer RNA (tRNA) genes, two ribosomal RNA (rRNA) genes, and a control region (CR). Most of the PCGs had typical ATN start codons and terminated with TAA or a single T residue. UUA (Leu2), AUU (Ile), UUU (Phe), AUA (Met) and AAU (Asn) were the five most frequently used codons. All tRNAs were folded into cloverleaf secondary structure, except for trnS1, which lacked the DHU arm. Phylogenetic analyses within the superfamily Zygaenoidea were performed based on multiple datasets from mitochondrial genes. The results showed that the families Phaudidae, Limacodidae and Zygaenidae were respectively recovered as monophyly; *C. houshuaii* was clustered in a clade with nettle type larvae in Limacodidae.

## Introduction

Limacodidae, commonly known as slug-moths, is a large family of Lepidopteran insects in the superfamily Zygaenoidea^1,2^. Limacodidae currently includes more than 310 genera and about 1800 species distributed worldwide, and is especially species-rich in the tropical and subtropical areas^1–3^. Unlike common caterpillars, the larvae of Limacodids usually have extremely reduced legs, and instead they developed suckers, so while advancing, their ventral surfaces are in direct contact with the substrate and make slug-like peristaltic movements^1,2^. The larvae of most Limacodid species have bright conspicuous warning colors and bear diverse cuticular processes of various types on the dorsum. The processes can be in the forms of spiny scoli (nettle), hairy tubercules, verrucae, or gelatinous warts among different species^3,4^. In particular, the nettle ones can produce urticating toxins, such as histamine and histidine, that can cause painful stings and skin inflammations when touched inadvertently^5–8^. Many of the Limacodidae are notorious pests, as they damage all kinds of tropical economic crops, such as banana, tea, sugarcane, cocoa and palm trees^4,9–11^.

Nowadays, Limacodidae has been widely accepted as a valid family by most modern taxonomists, although there remain some minor debates about its boundary delimitation within Zygaenoidea^1,12–15^. In Limacodidae, no tribal level taxon has ever been formally proposed, other than several morphology-based generic complexes raised decades ago by Holloway^16^ and Epstein^1^ respectively. Furthermore, the validation of these complexes has never been strictly tested with molecular methods since their establishments. As Limacodidae expanded in species number, these complexes have become obsolete, with many newly discovered species not fitting in. Therefore, the tribal level phylogenetic analysis for Limacodidae has been urgently demanded. Unfortunately, only two molecular phylogenetic studies investigating into the evolution of larval traits within Limacodidae have been published in recent years^12,17^. In these studies, the resolution of six ‘lineages’ that partially agreed with the old generic complex system in Limacodidae was generated, but no high-level classification has been carried out. To date, the problems of the overall classification within Limacodidae remain unsettled.

The insect mitochondrial genome (mitogenome) is a compact double-stranded circular DNA molecule, in the size typically varying between 15 and 18 kb, and usually harbors a set of 37 genes (13 protein-coding genes, 22 transfer RNA genes and two ribosomal RNA genes), which is conserved across bilaterian metazoans^18,19^. In addition to the genes, a mitogenome usually at least includes one A + T-rich noncoding control region of various lengths, which is responsible for the regulation in transcription and replication^19,20^. The small size and high cellular abundance of the mitogenome make it relatively much easier to obtain than nuclear genome^19,21^. In fact, the sequence data of insect mitogenomes are being accumulated rapidly in recent years, benefiting from the development of the next generation sequencing (NGS) technology, and meanwhile, used extensively in evolutionary studies, especially in phylogenetics^19,22,23^.

To date, there are only 21 complete or near complete mitochondrial genome sequences published for Limacodidae, covering mere 16 species in 13 genera (up to 31 December 2023). That is too small a proportion for such a large family as Limacodidae that consists of hundreds of genera and thousands of species. Therefore, in this study, we reported the first complete mitochondrial genome of the genus *Chibiraga*. *Chibiraga* is a mall East Asian Limacodid genus that includes three known species (*C. banghaasi*, *C. houshuaii* and *C. yukei*). All the adults of the genus are middle-sized plain-looking brown moths with darker markings on the forewings. The type species *C. banghaasi* is widely distributed in China, Korea and Russia. Its larvae are of the nettle-type, yellow in color, with blue-black markings and a bright-colored eye spot on dorsum, and they feed on Burseraceae and Cupressaceae plants^24^. *C. houshuaii* is distributed in Yunnan and Henan Provinces, China. *C. yukei* is in Sichuan Province, China. The larvae of the latter two species are yet unknown. The mitogenome was sequenced from *C. houshuaii*. We described and analyzed the mitogenome in details. we also further analyzed the phylogenetic relationships of three families in Zygaenoidea and explored the phylogenetic location of *C. houshuaii*. The establishment of this genome resource will provide insights for future relevant taxonomic and phylogenetic researches.

## Results

### Mitogenome structure and organization.

**Figure 1:**
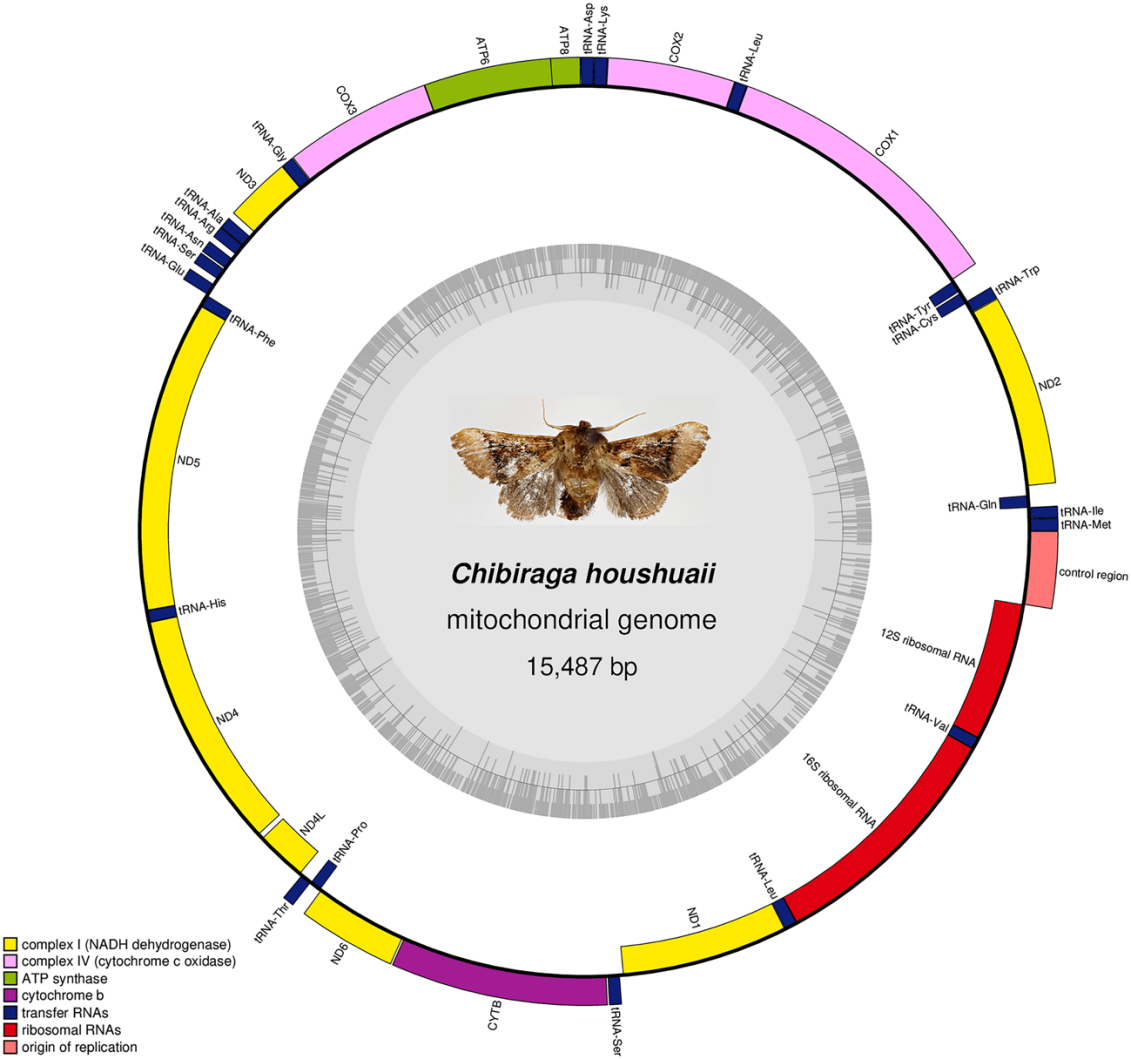
The circular structure of the mitogenome of *C. houshuaii*. Genes shown on the inner circle are transcribed in a clockwise direction, whereas those on the outer circle are transcribed counterclockwise. Different gene types are shown as filled boxes in different colors. ND1-6 are indicated in yellow; COX1-3 in light purple; ATP6 and 8 in green; CYTB in purple; tRNA genes in blue; rRNA genes in red and the control region in pink.

The complete mitogenome of *C. houshuaii* was a double-stranded circular DNA molecule of 15,487 bp in length (GenBank: OR619569). It shared the typical composition of 37 genes (13 PCGs, 22 tRNA genes and two rRNA genes) and an A + T-rich control region with other metazoan animals^25^ (Fig. 1). The gene arrangement pattern of this mitogenome followed that of the other Ditrysian moths^26,27^, with nine PCGs and 14 tRNAs encoded on the majority (J) strand, while the remaining four PCGs, eight tRNAs and the two rRNAs on the minority (N) strand (Table 1).

**Table 1:** Annotation of the mitochondrial genome of *C. houshuaii*.

Gene	Position	Size (bp)	Start/stop codon	Anticodon	Intergenic nucleotides	Strand
*trnM*	1–68	68		CAU	0	J
*trnI*	69–132	64		GAU	0	J
*trnQ*	130–198	69		UUG	− 3	N
*ND2*	251–1264	1014	ATT/TAA		52	J
*trnW*	1267–1333	67		UCA	2	J
*trnC*	1326–1391	66		GCA	− 8	N
*trnY*	1402–1469	68		GUA	10	N
*COX1*	1477–3007	1531	CGA/T		7	J
*trnL2*	3008–3074	67		UAA	0	J
*COX2*	3075–3750	676	ATA/T		0	J
*trnK*	3751–3821	71		CUU	0	J
*trnD*	3822–3892	71		GUC	0	J
*ATP8*	3893–4051	159	ATC/TAA		0	J
*ATP6*	4045–4722	678	ATG/TAA		− 7	J
*COX3*	4722–5507	786	ATG/TAA		− 1	J
*trnG*	5510–5577	68		UCC	2	J
*ND3*	5578–5931	354	ATT/TAA		0	J
*trnA*	5965–6028	64		UGC	33	J
*trnR*	6029–6091	63		UCG	0	J
*trnN*	6122–6187	66		GUU	30	J
*trnS1*	6196–6261	66		GCU	8	J
*trnE*	6297–6363	67		UUC	35	J
*trnF*	6368–6435	68		GAA	4	N
*ND5*	6435–8183	1749	ATT/TAA		− 1	N
*trnH*	8184–8249	66		GUG	0	N
*ND4*	8250–9588	1339	ATG/T		0	N
*ND4L*	9617–9904	288	ATG/TAA		28	N
*trnT*	9917–9981	65		UGU	12	J
*trnP*	9982–10,046	65		UGG	0	N
*ND6*	10,052–10,576	525	ATA/TAA		5	J
*CYTB*	10,585–11,736	1152	ATG/TAA		8	J
*trnS2*	11,744–11,810	67		UGA	7	J
*ND1*	11,829–12,767	939	ATG/TAA		18	N
*trnL1*	12,769–12,841	73		UAG	1	N
*rrnL*	12,842–14,231	1390			0	N
*trnV*	14,232–14,298	67		UAC	0	N
*rrnS*	14,299–15,081	783			0	N
Control region	15,082–15,487	406			0	J

The mitogenome of *C. houshuaii* exhibited a distinct overall composition bias towards A + T nucleotides (79.8%). This predominance of A + T content varied slightly among different types of genes. It was most significant in the control region (90.1%), followed by rRNAs (84.4%), tRNAs (82.0%), and least in PCGs (78.0%) (Table 2). The AT and GC-skew values were used to describe the compositional bias between the two DNA strands^28^. In this study, no great AT-skew had been detected either over the entire sequence or in the different genic regions (− 0.02 to 0.04) (Table 2). Nonetheless, the GC-skew was significant among the whole genome. The GC-skew values were mostly negative (− 0.19 to − 0.13), and positive only in the rRNA gene regiones (0.36) (Table 2).

**Table 2:** Base composition in different regions of the mitochondrial genome of *C. houshuaii*.

Feature	Size (bp)	A%	C%	G%	T%	A + T%	AT-skew	GC-skew
Whole genome	15,487	39.0	12.0	8.2	40.8	79.8	− 0.02	− 0.19
PCGs	11,182	38.1	12.8	9.2	39.9	78.0	− 0.02	− 0.16
tRNA genes	1465	41.5	10.2	7.8	40.5	82.0	0.01	− 0.13
rRNA genes	2173	43.9	5.0	10.6	40.5	84.4	0.04	0.36
Control region	406	44.5	5.7	4.2	45.6	90.1	− 0.01	− 0.15

In the mitogenome of *C. houshuaii*, there were 17 spacer regions of 1–52 bp between adjacent genes, in addition to five gene overlaps of 1–8 bp (Table 1). The longest spacer appeared between *trnQ*-*ND2* genes, which was conservative among all lepidopterans. The largest overlap was located between *trnW* and *trnC* genes.

### PCGs and codon usage.

The total length of the 13 PCGs in the mitogenome of *C. houshuaii* was 11,182 bp, accounting for 72.2% of the entire sequence. Most PCGs of *C. houshuaii* used typical start codon ATN at initiation (six with ATG, three with ATT, two with ATA, and one with ATC), only *COX1* started with an unorthodox putative codon CGA (Table 1). In respect to stop codons, *COX1*, *COX2* and *ND4* used a single T residue to stop transcription, all other ten genes terminated with the regular stop codon TAA (Table 1).

**Figure 2:**
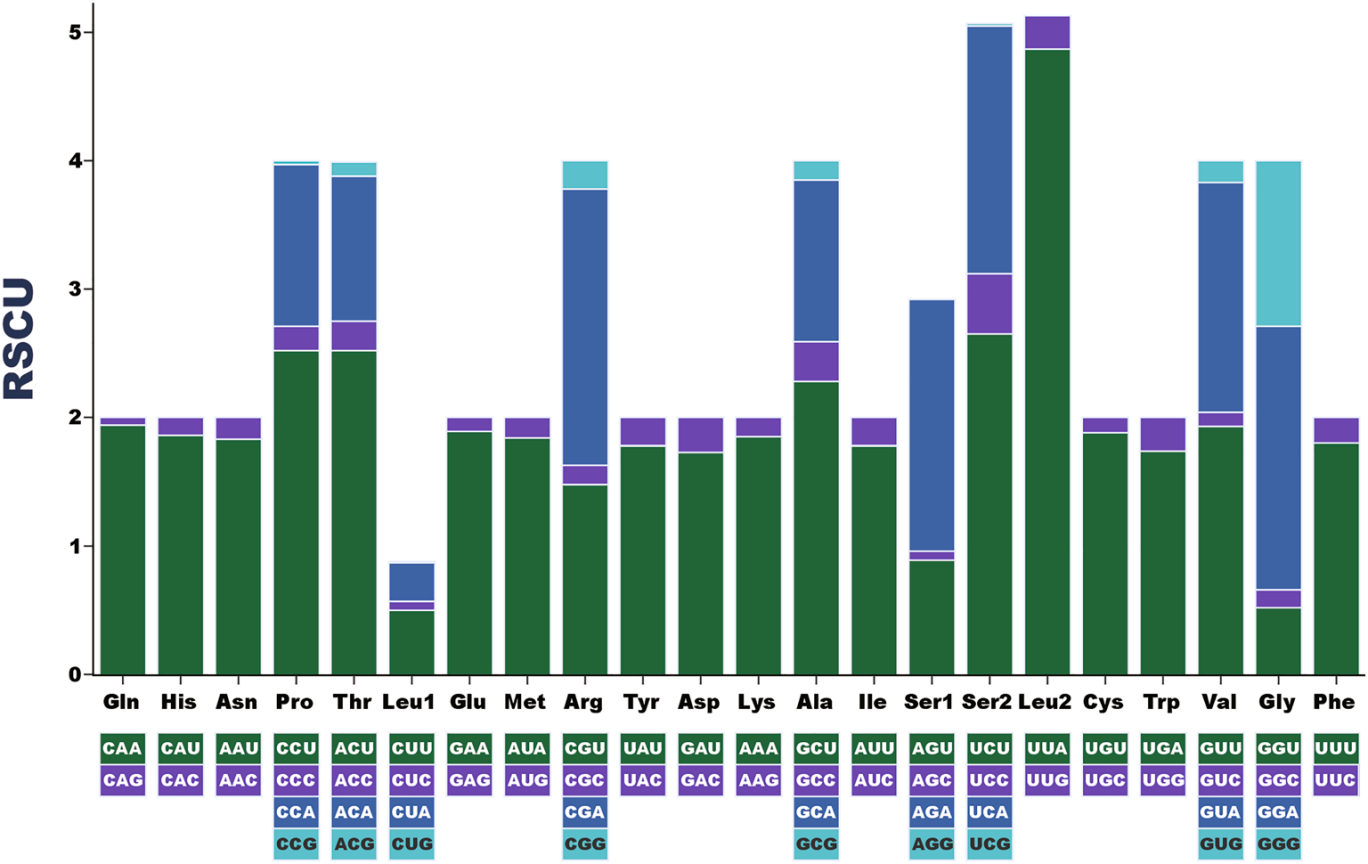
Relative synonymous codon usage (RSCU) of the PCGs in the *C. houshuaii* mitogenome.

The calculation of the relative synonymous codon usage (RSCU) of *C. houshuaii* indicated that the five most frequently used codons were UUA (Leu2), AUU (Ile), UUU (Phe), AUA (Met) and AAU (Asn), having the counts of 438, 384, 360, 261 and 238, while the least commonly used codons were CCG (Pro), UCG (Ser2), CUG (Leu1), each having only one count, and CGC (Arg), UGC (Cys), CAG (Gln), each having two counts. In conclusion, the synonymous codons ending with A or T were distinctly more preferably used than those ending with C or G, when coding the same amino acid (Fig. 2).

**Figure 3:**
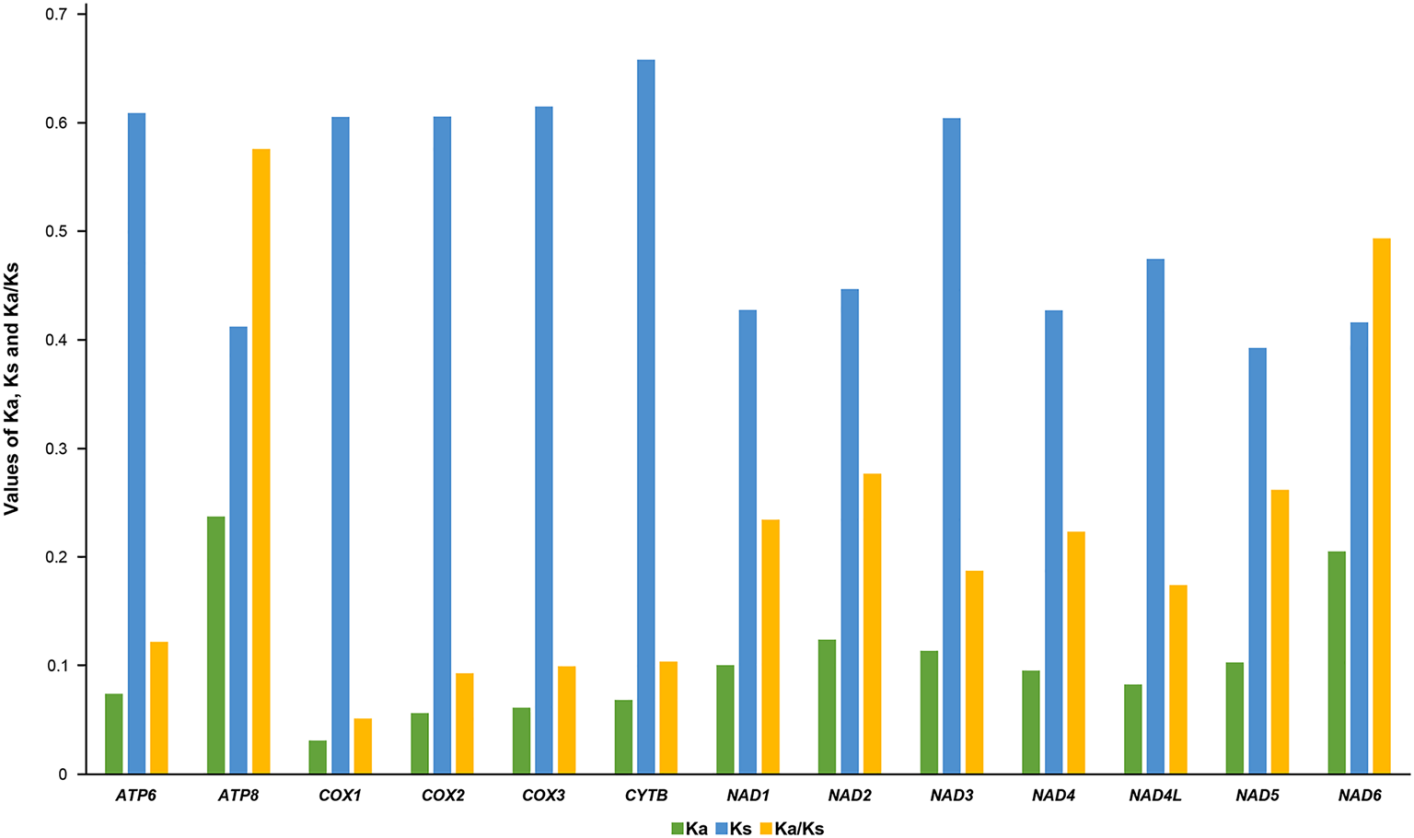
The Ka, Ks, and Ka/Ks values of the 13 mitochondrial PCGs in Limacodidae.

In addition, the synonymous substitutions (Ks), non-synonymous substitutions (Ka), and the Ka/Ks ratios of the 13 PCGs from Limacodidae were calculated (Fig. 3). The Ka/Ks values were used to estimate the natural selection patterns for the PCGs among a population^29^. In our study, the Ka/Ks values of all 13 PCGs were less than 1 (0.05–0.6), indicating the presence of purifying selection in these Limacodidae species.

### tRNA genes, rRNA genes, and the control region.

The total length of the 22 transfer RNA genes of *C. houshuaii* was 1465 bp, accounting for 9.5% of the entire mitogenome. Their respective lengths ranged from the shortest *trnR* with 63 bp to the longest *trnL1* with 73 bp (Table 1). The predicted secondary structures were shown in Supplementary Fig. S1. All tRNAs could be folded into the iconic cloverleaf secondary structure, except for trnS1, which did not have the typical DHU arm, but a single-stranded loop instead. A total of 19 wobbled G-U pairs (six on acceptor arms, six on DHU arms, six on anticodon arms, and one on TΨC arms), that formed weak bonds were found in 13 tRNAs. In addition, the atypical U-U pairing was also detected, but only in trnL2, trnP and trnS2.

The total length of the two ribosomal RNA genes (16S rRNA = 1390 bp; 12S rRNA = 783 bp) was 2173 bp, accounting for 14.0% of the entire mitogenome (Table 1). The 16S rRNA gene was located between *trnL1* and *trnV*, and the 12S rRNA gene was between *trnV* and the control region, both without intergenic spacers or overlaps. The predicted secondary structures of the two rRNAs were shown in Supplementary Figs. S2 and S3 respectively. There were five domains (I, II, IV, V, VI) in the 16S rRNA of *C. houshuaii*, and the domain III was absent as in all Arthropoda species^30^. There were three domains (I, II, III) in the 12S rRNA. Many mismatches, such as G-U, G-A, or U-U pairs were observed in Both rRNAs.

**Figure 4:**
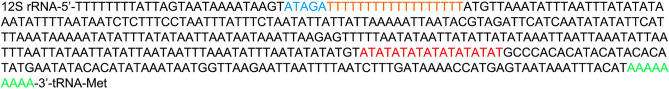
The control region in mitochondrial genome of *C. houshuaii*.

The control region of *C. houshuaii* was 406 bp in length, and was located between the 12S rRNA and *trnM* genes (Table 1). The following transcription or replication related elements were identified (Fig. 4): an ATAGA motif, followed by an 18 bp polyT stretch located at downstream of 12S rRNA; a 9 bp polyA stretch at the upstream of *trnM*; and a microsatellite-like repeat region (AT)_9_. However, no tandem repeat sequence or any functional stem-loop structure was found.

### Phylogenetic analyses.

**Figure 5:**
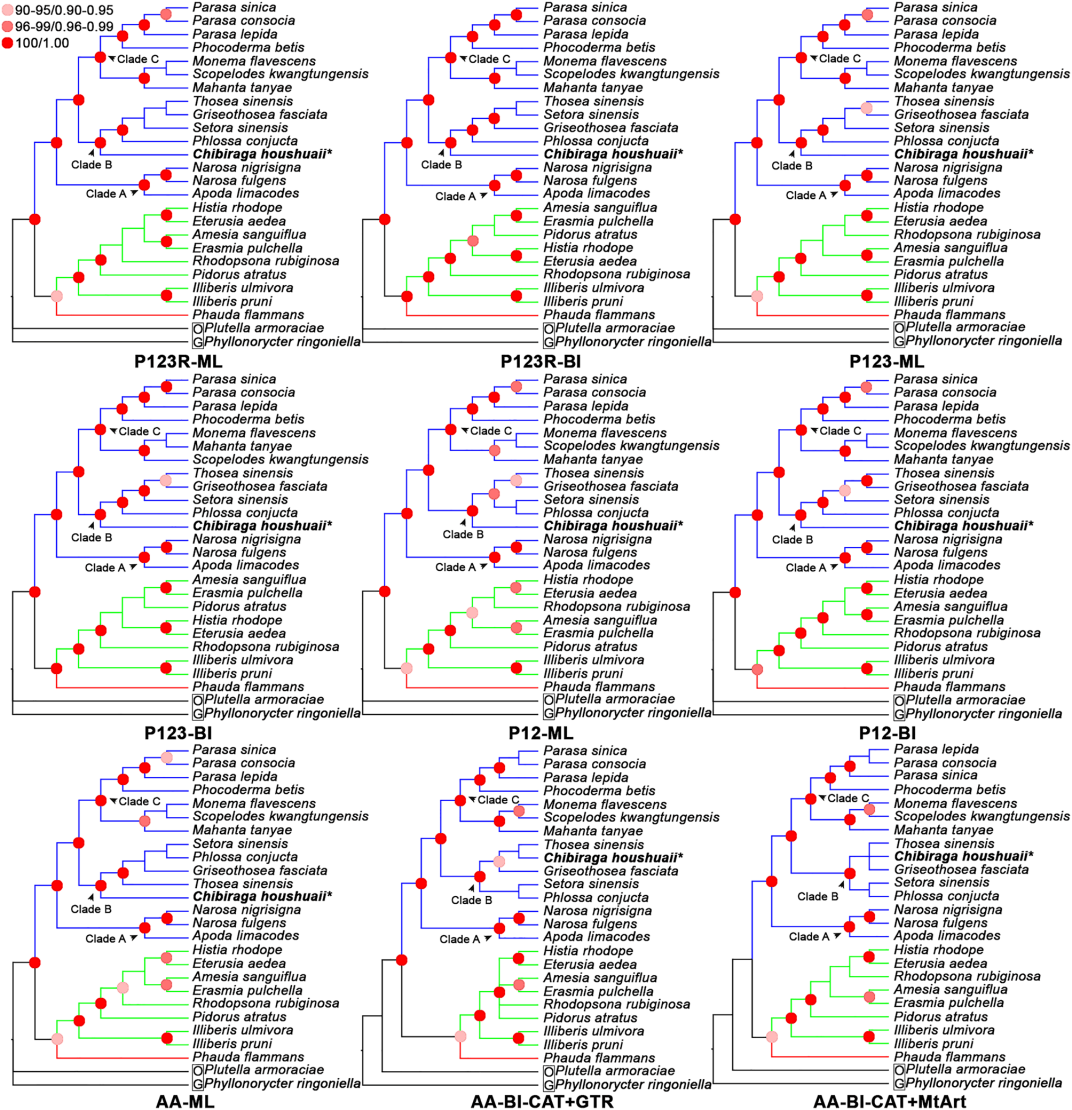
Inferred phylogenetic relationships among Zygaenoidea from the four datasets using different methods and models. Branch in red represents family Phaudidae; branches in green represent family Zygaenidae; branches in blue represent family Limacodidae. Nodes with 90–95 bootstrap support values in ML analyses or 0.90–0.95 posterior probabilities in BI analyses are labelled by light pink circles; 96–99 or 0.96–0.99 by pink circles; 100 or 1.00 by red circles. The species followed by an asterisk is sequenced for this study. “OG” in the black box is short for “outgroup”.

Different combinations of datasets, partitioning models and tree-building methods resulted in nine phylogenetic trees within Zygaenoidea (Fig. 5). Their topologies are largely congruent, especially on family and tribal levels. In all nine trees, the three families (Phaudidae, Limacodidae and Zygaenidae) appeared in our analyses were successfully recovered as monophylies in the superfamily Zygaenoidea with strong nodal supports. The sibling relationship between Phaudidae and Zygaenidae was highly supported, and together being the sister group to Limacodidae. This backbone topology was consistent with other recent phylogenetic analyses concerning Zygaenoidea^21,31^. Limacodidae was resolved into three major clades, Clade A, B and C, which roughly equaled to the Lineage 3, 5 and 6 respectively in Lin et al.^17^. Our species *C. houshuaii* was clustered into Clade B, where all the member Limacodids had nettle type larvae. Noteworthily, *C. houshuaii* was consistently recovered at the base of Clade B with strong nodal supports in all seven trees produced using site-homogeneous substitution models, regardless of the analytical methods and datasets used. However, in the two BI trees (AA-BI-CAT + GTR and AA-BI-CAT + MtArt) produced from site-heterogeneous models, *C. houshuaii* was positioned much more terminally, being sibling to *Thosea sinensis*, only with weak node confidence.

Although all these trees were identical in the high-level backbone topologies, there were still minor discrepancies among them within each family or major clade. Such as mentioned above, *C. houshuaii* was located either at the base or terminal of Clade B, depending on the type of the substitution models used. The positionings of *Rhodopsona rubiginosa* and *Pidorus atratus* were quite unstable as well. In four trees (P123R-ML, P12-BI, AA-ML and AA-BI-CAT + GTR), they were located at more basal positions in Zygaenidae, while in other trees, either of them was located at more terminal positions (*Rhodopsona rubiginosa* in three trees: P123-ML, P12-ML and AA-BI-CAT + MtArt; *Pidorus atratus* in two trees: P123R-BI and P123-BI).

## Discussion

In this study, the phylogenetic results confirmed the monophylies of Phaudidae, Limacodidae and Zygaenidae. The phylogeny within these three families was Limacodidae + (Phaudidae + Zygaenidae), supporting the results in other mitochondrial genes-based phylogenetic studies^21,31^. Although, Limacodidae was recovered as monophyletic, two closely related problematic families Chrysopolomidae and Dalceridae had not been included in the present analyses, due to the scarcity of their mitogenomes. Chrysopolomidae and Dalceridae were two small families compared to Limacodidae. Epstein^1^, based on morphological observations, suggested the degradation of Chrysopolomidae to a subfamily in Limacodidae, and Dalceridae should be included within Limacodidae as well. In addition, two recent multi-gene phylogenetic studies also supported the inclusion of these two families within Limacodidae^12,14^. Because of the limited samplings of these two families in the above studies, it was immature to jump directly into any changes in their classification, and because of that, Chrysopolomidae and Dalceridae had maintained their familial status^13,15^. Obviously, many more mitochondrial genomes were demanded to be sequenced in order to better understand the monophyly of Limacodidae as well as its interior phylogeny.

## Methods

### Sample collection, DNA extraction and sequencing.

The adult specimen of *C. houshuaii* was collected by light trap from Mountain Jizu, Dali Bai Ethnic Autonomous Prefecture, Yunnan Province, China (25° 56′ 37′′N, 100° 23′′ 14′′ E) on 6 August 2021. The specimen was then deposited in 95% ethanol under − 20 °C in the Insect Collection of Guizhou University of Traditional Chinese Medicine, Guiyang, China, until DNA extraction. The total DNA was extracted from two legs using a MagicMag Genomic DNA Micro Kit (Sangon Biotech Co., Shanghai, China) according to the manufacturer’s protocol. A total amount of 0.2 μg sample DNA was fragmented by sonication to a size of 350 bp, then subjected to high-throughput pair-ended sequencing (PE150) on Illumina 6000 platform in Novogene Bioinformatics Technology Co., Ltd (Tianjin, China).

### Mitogenome assembly, annotation, and analyses.

The raw data were processed using Fastp V.0.19.7^32^ to discard low-quality reads and obtain a high-quality clean data of 3.26 g bp. The complete mitogenome was de novo assembled by MitoZ V.3.4^33^ and SPAdes V.3.15.1^34^. The sequence was polished with the aid of Pilon V.1.24^35^, then annotated using MitoZ software and MITOS WebServer (http://mitos2.bioinf.uni-leipzig.de/index.py)^36^, and subsequently manually checked. The final mitogenome map was produced using Organellar Genome DRAW (OGDRAW)^37^.

The statistics of the mitogenome base composition was produced in MEGA V11.0.11^38^. The asymmetry of the two mitogenome strands was evaluated using the formulae reported by Perna and Kocher^28^: AT-skew = (A− T)/(A + T), and GC-skew = (G− C)/(G + C). The relative synonymous codon usage (RSCU) of the PCGs was analyzed and visualized in PhyloSuite V.1.2.3^39,40^. The non-synonymous substitutions (Ka), synonymous substitutions (Ks), and their ratios (Ka/Ks) for the 13 PCGs in Limacodidae were calculated through DnaSP V.5.10.01^41^. The secondary structures of the tRNAs were predicted on MITOS WebServer. The secondary structures of the rRNAs were predicted with R2DT V.1.3^42^. Tandem repeat elements in the control region were detected by using Tandem Repeats Finder (https://tandem.bu.edu/trf/home)^43^, and the possible helix structures were predicted on Mfold Web Server (http://www.unafold.org/mfold/applications/dna-folding-form.php)^44^.

### Phylogenetic analyses.

We chose 24 taxa, including our newly sequenced one, from three families in the superfamily Zygaenoidea as ingroup, and two other taxa (*Phyllonorycter ringoniella* and *Plutella armoraciae* as outgroup) from Gracillarioidea and Yponomeutoidea respectively to analyze the phylogenetic position of *C. houshuaii*, and the phylogenetic relationships within Zygaenoidea (Table 3). In these analyses, a total of nine Maximum Likelihood (ML) or Bayesian Inference (BI) trees were reconstructed based on four data matrixes, (1) “P123R” matrix: all three positions of 13 PCGs plus two rRNA genes (12,863 sites); (2) “P123” matrix: all three positions of 13 PCGs (11,118 sites); (3) “P12” matrix: 1st and 2nd positions of PCGs (7412 sites); (4) “AA” matrix: amino acid sequences translated from 13 PCGs (3706 sites). The DNA sequences of each gene were first aligned separately using MAFFT V.7.52^45^ with the L-INS-i algorithm, next trimmed by trimAI V.1.4.1^46^, then concatenated in MEGA 11 software, and last converted into the above-mentioned four datasets. Two site-heterogeneous substitution models (CAT + GTR and CAT + MtArt respectively) were used for the AA matrix in the BI analyses. The site-homogeneous models were used in all our other tree-building analyses, and the optimal partitioning scheme and the corresponding best fit substitution models for each analysis were determined using ModelFinder V.2.2.2^47^ implemented in IQ-TREE2 V.2.2.2.7^48^ (Supplementary Tables S1–S7). The ML trees were constructed in IQ-TREE2 software, and the node confidence values were assessed with 20,000 ultrafast bootstraps^49^. The BI trees from AA matrix were generated via PhyloBayes-MPI V.1.8c^50^. For each tree, two independent searches were run for 25 thousand generations until the maxdiff values dropped below 0.1. The BI trees from the other three datesets were generated via MrBayes V.3.2.7^51^ in parallel mode. For each tree, two independent searches were run for five million generations until the average standard deviations of split frequencies dropped below 0.01. In each BI analysis, the initial 25% trees of each run were discarded as burnin, then the consensus trees and the posterior probability (PP) of each node were computed from the remaining trees. The phylogenetic trees were visualized in FigTree V.1.4.4^52^.

**Table 3:** Basic information of the mitogenomes used in the phylogenetic analyses in this study.

Superfamily	Family	Taxa	Genbank accession number
Ingroup			
Zygaenoidea	Limacodidae	*Apoda limacodes*	OX291565
		*Chibiraga houshuaii*	**OR619569**
		*Griseothosea fasciata*	MK250437
		*Mahanta tanyae*	MK396080
		*Monema flavescens*	KU946971
		*Narosa fulgens*	OP919326
		*Narosa nigrisigna*	MH675969
		*Parasa consocia*	OK149235
		*Parasa lepida*	OP132386
		*Parasa sinica*	MK122617
		*Phlossa conjucta*	OP132387
		*Phocoderma betis*	OP919337
		*Scopelodes kwangtungensis*	OQ848600
		*Setora sinensis*	OP160524
		*Thosea sinensis*	MK122624
	Phaudidae	*Phauda flammans*	MN150296
	Zygaenidae	*Amesia sanguiflua*	MK224510
		*Erasmia pulchella*	OQ134124
		*Eterusia aedea*	MH316560
		*Histia rhodope*	MF542357
		*Illiberis pruni*	MZ726799
		*Illiberis ulmivora*	MT075808
		*Pidorus atratus*	MG882482
		*Rhodopsona rubiginosa*	KM244668
Outgroup			
Gracillarioidea	Gracillariidae	*Phyllonorycter ringoniella*	OM287125
Yponomeutoidea	Plutellidae	*Plutella armoraciae*	MW662613

Accession number of the newly sequenced species in bold.

### Supplementary Information

Supplementary Information.

## Data Availability

The genome sequence data that support the findings of this study are openly available in GenBank of NCBI at https://www.ncbi.nlm.nih.gov/nuccore/OR619569 under the Accession no. OR619569.
