# Supplementary material for: The complete mitochondrial genome of Chibiraga houshuaii (Lepidoptera, Limacodidae) and its phylogenetic implications
Source: Sci Rep. 2024 Mar 25;14:7009. doi: 10.1038/s41598-024-57709-4 (PMC10963781; doi:10.1038/s41598-024-57709-4)
Supplement: Supplementary file 1 — Supplementary Information. [file 41598_2024_57709_MOESM1_ESM.pdf]

Supplementary Information

# The complete mitochondrial genome of *Chibiraga houshuaii* (Lepidoptera, Limacodidae) and its phylogenetic implications

Yanpeng Cai and Aihui Yin

**Contents**

**Figure S1.** tRNA secondary structures of *C. houshuaii*.
**Figure S2.** The 16S rRNA secondary structure of *C. houshuaii*.
**Figure S3.** The 12S rRNA secondary structure of *C. houshuaii*.
**Table S1.** The partitioning scheme and corresponding substitution models determined by ModelFinder for the P123R dataset in the ML analysis. The p1, p2 and p3 indicate the first, second and third codon positions of each PCG respectively.
**Table S2.** The partitioning scheme and corresponding substitution models determined by ModelFinder for the P123R dataset in the BI analysis. The p1, p2 and p3 indicate the first, second and third codon positions of each PCG respectively.
**Table S3.** The partitioning scheme and corresponding substitution models determined by ModelFinder for the P123 dataset in the ML analysis. The p1, p2 and p3 indicate the first, second and third codon positions of each PCG respectively.
**Table S4.** The partitioning scheme and corresponding substitution models determined by ModelFinder for the P123 dataset in the BI analysis. The p1, p2 and p3 indicate the first, second and third codon positions of each PCG respectively.
**Table S5.** The partitioning scheme and corresponding substitution models determined by ModelFinder for the P12 dataset in the ML analysis. The p1 and p2 indicate the first and second codon positions of each PCG respectively.
**Table S6.** The partitioning scheme and corresponding substitution models determined by ModelFinder for the P12 dataset in the BI analysis. The p1 and p2 indicate the first and second codon positions of each PCG respectively.
**Table S7.** The partitioning scheme and corresponding substitution models determined by ModelFinder for the AA dataset in the ML analysis.

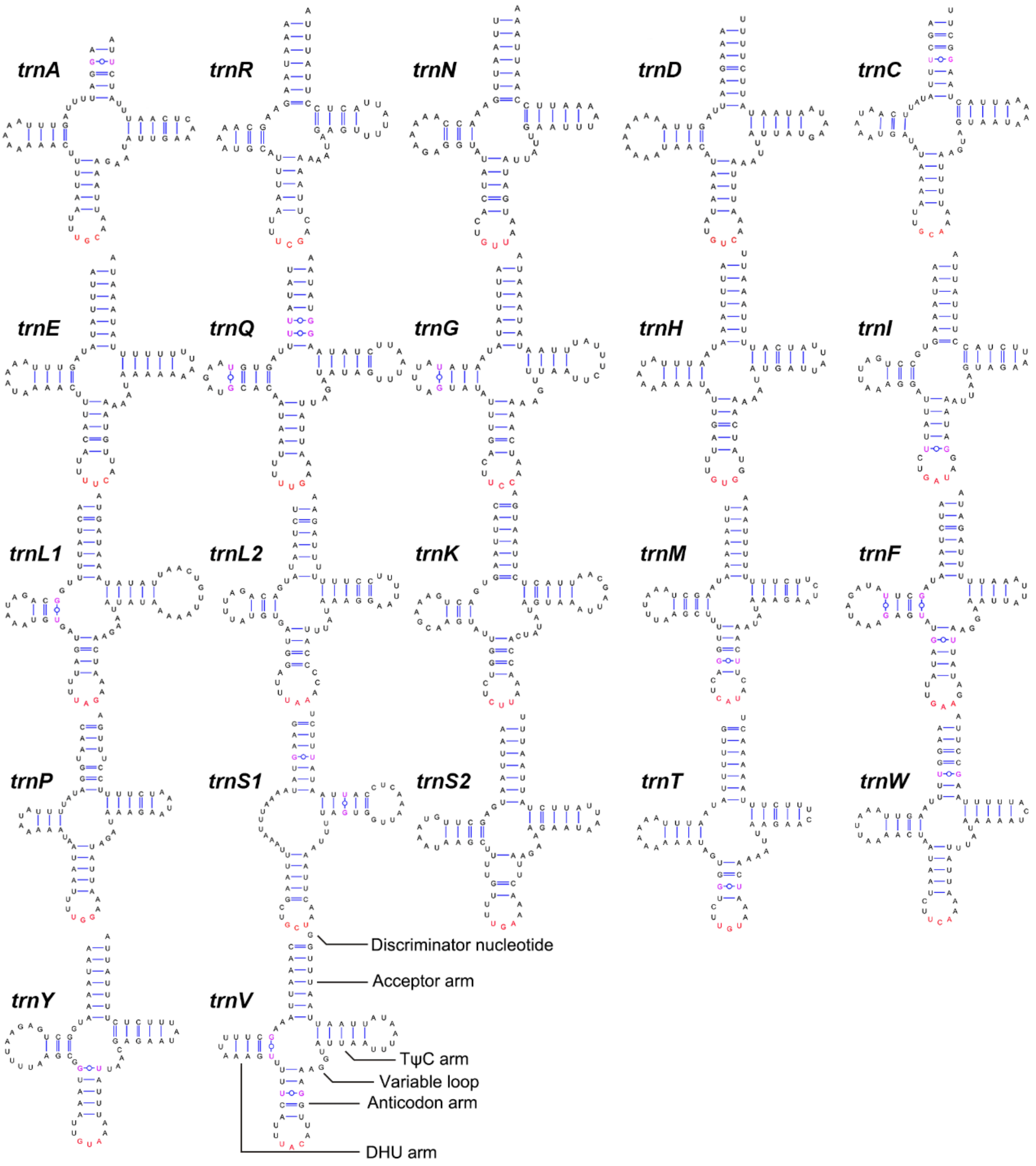


**Figure S1.** tRNA secondary structures of *C. houshuaii*.

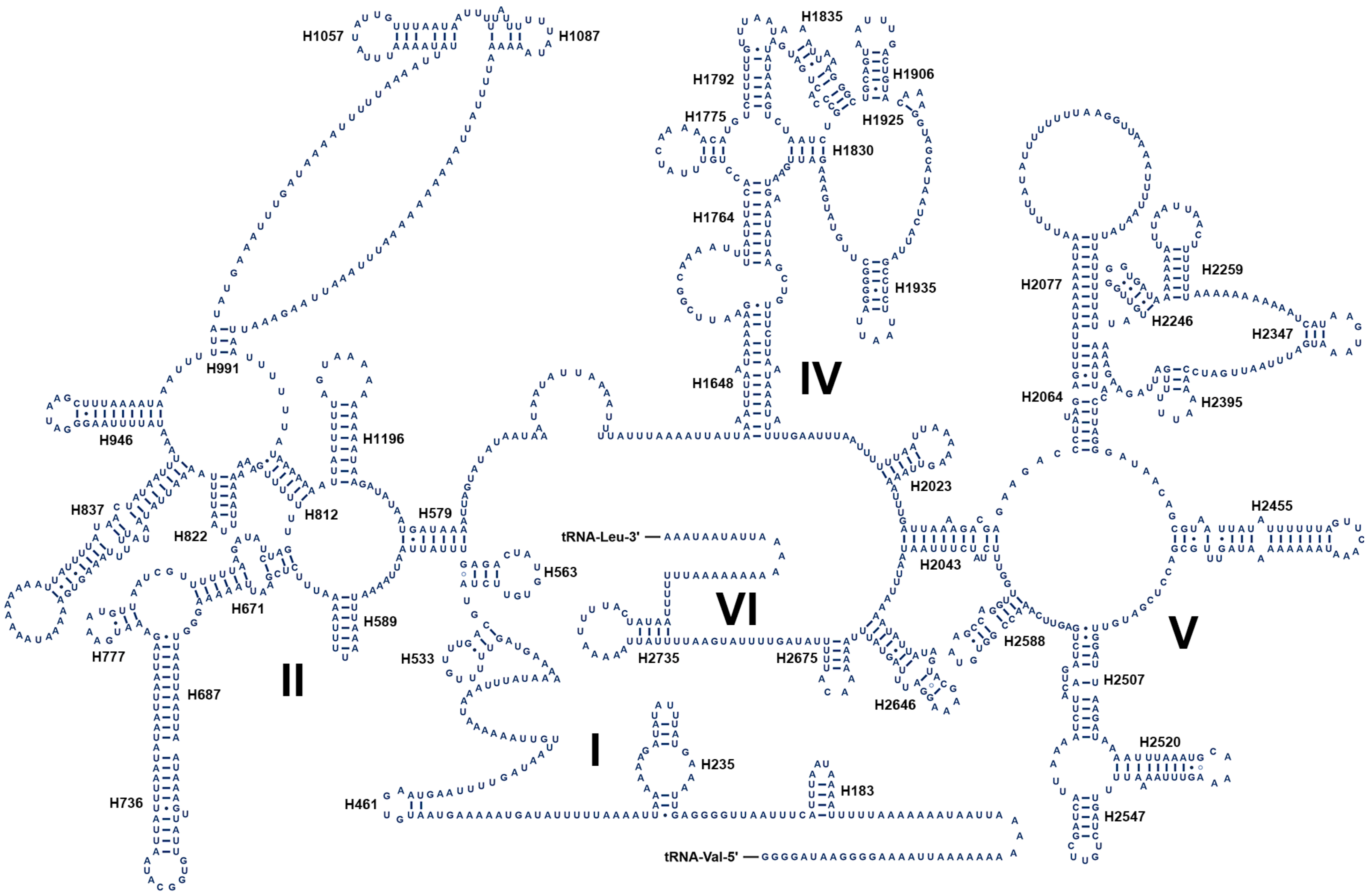


**Figure S2.** The 16S rRNA secondary structure of *C. houshuaii*.

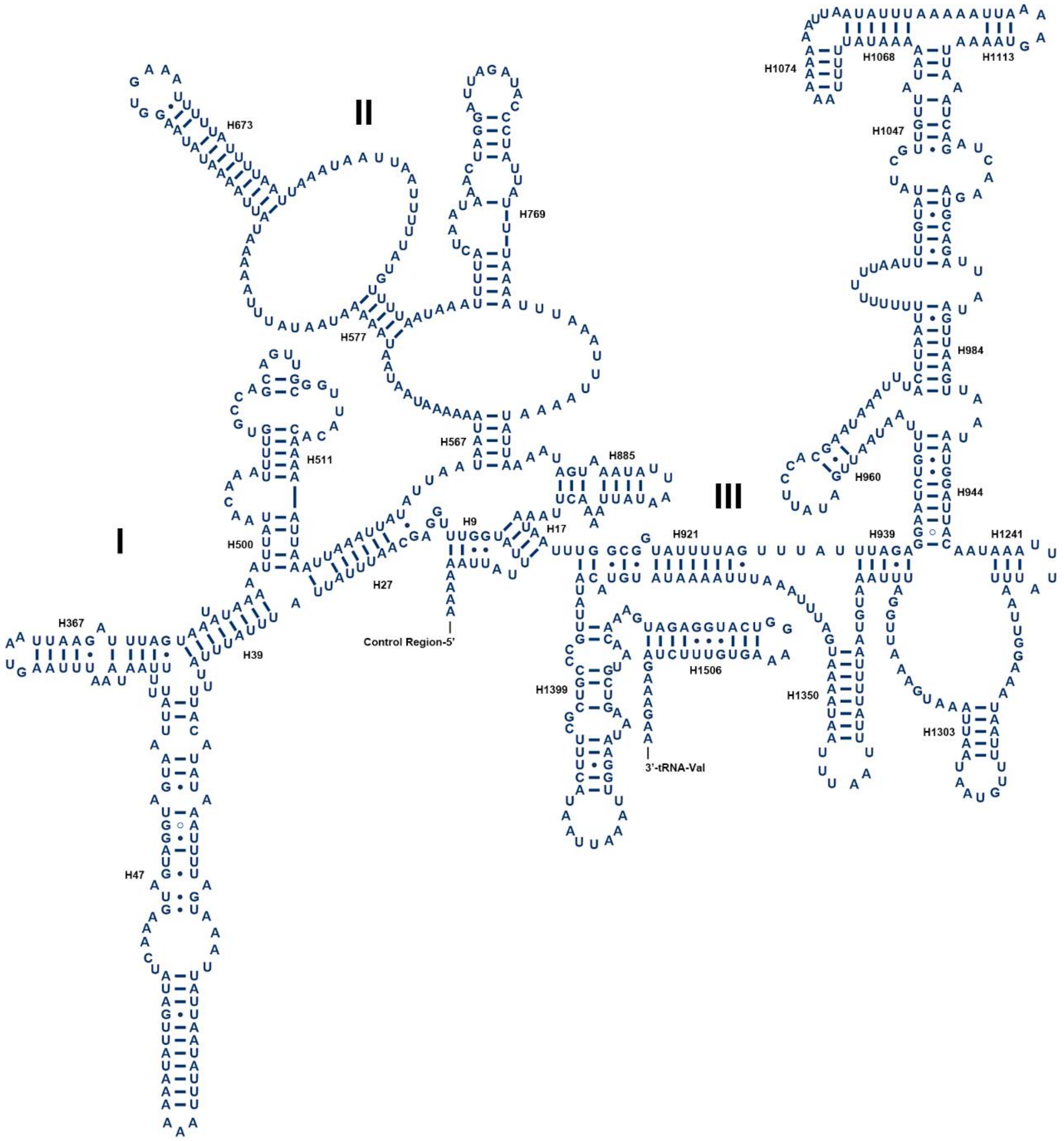


**Figure S3.** The 12S rRNA secondary structure of *C. houshuaii*.

| Partitions | Models | Data partitions |
|---|---|---|
| P1 | GTR+F+I+G4 | atp6p1, cox3p1, cytbp1, nd3p1 |
| P2 | TIM+F+G4 | atp6p3, atp8p3, cox1p3, cox2p3, cox3p3, cytbp3, nd2p3, nd3p3, nd6p3 |
| P3 | TVM+F+I+G4 | atp6p2, cox1p2, cox2p2, cox3p2, cytbp2 |
| P4 | TIM2+F+I+G4 | atp8p1, atp8p2, nd2p1, nd6p1 |
| P5 | TIM2+F+I+G4 | cox1p1, cox2p1 |
| P6 | TVM+F+G4 | nd1p1, nd4p1, nd4lp1, nd5p1 |
| P7 | TIM+F+I+G4 | nd1p3, nd4p3, nd4lp3, nd5p3 |
| P8 | TVM+F+I+G4 | nd1p2, nd4p2, nd4lp2, nd5p2 |
| P9 | TVM+F+I+G4 | nd2p2, nd3p2, nd6p2 |
| P10 | GTR+F+I+G4 | *rrnS*, *rrnL* |

**Table S1.** The partitioning scheme and corresponding substitution models determined by ModelFinder for the P123R dataset in the ML analysis. The p1, p2 and p3 indicate the first, second and third codon positions of each PCG respectively.

| Partitions | Models | Data partitions |
|---|---|---|
| P1 | GTR+F+I+G4 | *rrnS*, *rrnL* |
| P2 | GTR+F+I+G4 | atp6p1, cytbp1, nd3p1 |
| P3 | GTR+F+I+G4 | atp6p2, cox1p2, cox2p2, cox3p2, cytbp2 |
| P4 | GTR+F+G4 | atp6p3, cox1p3 |
| P5 | GTR+F+I+G4 | atp8p1, atp8p2, nd6p1 |
| P6 | GTR+F+G4 | atp8p3, cox2p3, cox3p3, cytbp3, nd3p3 |
| P7 | GTR+F+I+G4 | cox1p1, cox2p1, cox3p1 |
| P8 | GTR+F+G4 | nd1p1, nd4p1, nd4lp1, nd5p1 |
| P9 | GTR+F+I+G4 | nd1p2, nd4p2, nd4lp2, nd5p2 |
| P10 | GTR+F+I+G4 | nd1p3 |
| P11 | GTR+F+I+G4 | nd2p1 |
| P12 | GTR+F+I+G4 | nd2p2, nd3p2 |
| P13 | GTR+F+I+G4 | nd2p3 |
| P14 | GTR+F+I+G4 | nd4p3 |
| P15 | GTR+F+G4 | nd4lp3, nd5p3 |
| P16 | GTR+F+I+G4 | nd6p2 |
| P17 | GTR+F+I+G4 | nd6p3 |

**Table S2.** The partitioning scheme and corresponding substitution models determined by ModelFinder for the P123R dataset in the BI analysis. The p1, p2 and p3 indicate the first, second and third codon positions of each PCG respectively.

| Partitions | Models | Data partitions |
|---|---|---|
| P1 | GTR+F+I+G4 | atp6p1, cox3p1, cytbp1, nd3p1 |
| P2 | TIM+F+G4 | atp6p3, cox1p3, cox2p3, cox3p3, cytbp3, nd3p3 |
| P3 | TVM+F+I+G4 | atp6p2, cox1p2, cox2p2, cox3p2, cytbp2 |
| P4 | TIM2+F+I+G4 | atp8p1, atp8p2, nd2p1, nd6p1 |
| P5 | TIM+F+G4 | atp8p3, nd2p3, nd6p3 |
| P6 | TIM2+F+I+G4 | cox1p1, cox2p1 |
| P7 | TVM+F+I+G4 | nd1p1, nd4p1, nd4lp1, nd5p1 |
| P8 | TIM+F+I+G4 | nd1p3, nd4p3, nd4lp3, nd5p3 |
| P9 | TVM+F+I+G4 | nd1p2, nd4p2, nd4lp2, nd5p2 |
| P10 | TVM+F+I+G4 | nd2p2, nd3p2, nd6p2 |

**Table S3.** The partitioning scheme and corresponding substitution models determined by ModelFinder for the P123 dataset in the ML analysis. The p1, p2 and p3 indicate the first, second and third codon positions of each PCG respectively.

| Partitions | Models | Data partitions |
|---|---|---|
| P1 | GTR+F+I+G4 | atp6p1, cytbp1, nd3p1 |
| P2 | GTR+F+I+G4 | atp6p2, cox1p2, cox2p2, cox3p2, cytbp2 |
| P3 | GTR+F+G4 | atp6p3, atp8p3, cox1p3, cox2p3, nd3p3 |
| P4 | GTR+F+I+G4 | atp8p1, atp8p2, nd2p1, nd6p1 |
| P5 | GTR+F+I+G4 | cox1p1, cox2p1, cox3p1 |
| P6 | GTR+F+G4 | cox3p3, cytbp3 |
| P7 | GTR+F+G4 | nd1p1, nd4p1, nd4lp1, nd5p1 |
| P8 | GTR+F+I+G4 | nd1p2, nd4p2, nd4lp2, nd5p2 |
| P9 | GTR+F+G4 | nd1p3 |
| P10 | GTR+F+I+G4 | nd2p2, nd3p2, nd6p2 |
| P11 | GTR+F+G4 | nd2p3 |
| P12 | HKY+F+I+G4 | nd4p3 |
| P13 | GTR+F+G4 | nd4lp3, nd5p3 |
| P14 | HKY+F+G4 | nd6p3 |

**Table S4.** The partitioning scheme and corresponding substitution models determined by ModelFinder for the P123 dataset in the BI analysis. The p1, p2 and p3 indicate the first, second and third codon positions of each PCG respectively.

| Partitions | Models | Data partitions |
|---|---|---|
| P1 | GTR+F+I+G4 | atp6p1, cox3p1, cytbp1, nd3p1 |
| P2 | TVM+F+I+G4 | atp6p2, cox1p2, cox2p2, cox3p2, cytbp2 |
| P3 | TIM2+F+I+G4 | atp8p1, atp8p2, nd2p1, nd6p1 |
| P4 | TIM2+F+G4 | cox1p1, cox2p1 |
| P5 | TVM+F+I+G4 | nd1p1, nd4p1, nd4lp1, nd5p1 |
| P6 | TVM+F+I+G4 | nd1p2, nd4p2, nd4lp2, nd5p2 |
| P7 | TVM+F+I+G4 | nd2p2, nd3p2, nd6p2 |

**Table S5.** The partitioning scheme and corresponding substitution models determined by ModelFinder for the P12 dataset in the ML analysis. The p1 and p2 indicate the first and second codon positions of each PCG respectively.

| Partitions | Models | Data partitions |
|---|---|---|
| P1 | GTR+F+G4 | atp6p1, cox2p1, cox3p1, nd6p2 |
| P2 | GTR+F+I+G4 | atp6p2, cox1p2, cox2p2, cox3p2, cytbp2 |
| P3 | GTR+F+I+G4 | atp8p1, atp8p2, nd6p1 |
| P4 | GTR+F+G4 | cox1p1 |
| P5 | GTR+F+I+G4 | cytbp1, nd3p1 |
| P6 | GTR+F+I+G4 | nd1p1, nd4p1, nd4lp1, nd5p1 |
| P7 | GTR+F+I+G4 | nd1p2, nd4p2, nd4lp2, nd5p2 |
| P8 | GTR+F+I+G4 | nd2p1 |
| P9 | GTR+F+I+G4 | nd2p2, nd3p2 |

**Table S6.** The partitioning scheme and corresponding substitution models determined by ModelFinder for the P12 dataset in the BI analysis. The p1 and p2 indicate the first and second codon positions of each PCG respectively.

| Partitions | Models | Data partitions |
|---|---|---|
| P1 | mtMet+F+I+G4 | atp6, atp8, nd2, nd3, nd6 |
| P2 | mtART+F+I+G4 | cox1, cox2, cox3, cytb |
| P3 | mtMet+F+I+G4 | nd1, nd4, nd4l, nd5 |

**Table S7.** The partitioning scheme and corresponding substitution models determined by ModelFinder for the AA dataset in the ML analysis.
